# Antinociceptive effect of selective G protein-gated inwardly rectifying K^+^ channel agonist ML297 in the rat spinal cord

**DOI:** 10.1371/journal.pone.0239094

**Published:** 2020-09-11

**Authors:** Masami Kimura, Hiroaki Shiokawa, Yuji Karashima, Makoto Sumie, Sumio Hoka, Ken Yamaura

**Affiliations:** 1 Department of Anesthesiology and Critical Care Medicine, Graduate School of Medical Sciences, Kyushu University, Fukuoka, Japan; 2 Hayashi Eye Hospital, Fukuoka, Japan; 3 Operating Rooms, Kyushu University Hospital, Fukuoka, Japan; 4 International University of Health and Welfare, Graduate School of Medicine, Fukuoka, Japan; Tokyo Metropolitan Institute of Medical Science, JAPAN

## Abstract

The G protein-gated inwardly rectifying K^+^ (GIRK) channels play important signaling roles in the central and peripheral nervous systems. However, the role of GIRK channel activation in pain signaling remains unknown mainly due to the lack of potent and selective GIRK channel activators until recently. The present study was designed to determine the effects and mechanisms of ML297, a selective GIRK1/2 activator, on nociception in the spinal cord by using behavioral studies and whole-cell patch-clamp recordings from substantia gelatinosa (SG) neurons. Rats were prepared for chronic lumber catheterization and intrathecal administration of ML297. The nociceptive flexion reflex was tested using an analgesy-meter, and the influence on motor performance was assessed using an accelerating rotarod. We also investigated pre- and post-synaptic actions of ML297 in spinal cord preparations by whole-cell patch-clamp recordings. Intrathecal administration of ML297 increased the mechanical nociceptive threshold without impairing motor function. In voltage-clamp mode of patch-clamp recordings, bath application of ML297 induced outward currents in a dose-dependent manner. The ML297-induced currents demonstrated specific equilibrium potential like other families of potassium channels. At high concentration, ML297 depressed miniature excitatory postsynaptic currents (mEPSCs) but not their amplitude. The ML297-induced outward currents and suppression of mEPSCs were not inhibited by naloxone, a μ-opioid receptor antagonist. These results demonstrated that intrathecal ML297 showed the antinociceptive effect, which was mediated through direct activation of pre- and post-synaptic GIRK channels. Selective GIRK channel activation is a promising strategy for the development of new agents against chronic pain and opioid tolerance.

## Introduction

The G protein-gated inwardly rectifying K^+^ (GIRK) channels are potassium-selective ion channels that act as effectors of G_i/o_-coupled G protein-coupled receptors (GPCRs). Thus, increased GIRK channel activity leads to reduced cellular excitability making GIRK channels as one of the most important players in the physiological system. There are four distinct mammalian genes that encode the GIRK channels (termed GIRK1–GIRK4 or Kir3.1–Kir3.4); neuronal GIRK channels are comprised of either homotetramers or heterotetramers containing the GIRK1–GIRK3 subunits [1]. In the central and peripheral nervous systems, GIRK channels are mainly involved in postsynaptic inhibitory signaling [2, 3]. In the spinal cord, GPCRs such as opioid, adrenergic, muscarinic, and dopaminergic receptors, are involved in the regulation of pain transmission [4]. Therefore, GIRK channels in the spinal cord are probably valuable therapeutic targets for pain management.

It has been reported that ethanol directly activates several subtypes of GIRK channels without the involvement of G proteins or second messengers [5]. However, ethanol exerts its effects on many channels, including NMDA and GABA_A_ receptors, and therefore, it is not a specific GIRK activator. The lack of a selective GIRK channel agonist had prevented the exploration of the therapeutic potential of GIRK channel activation until 2013 when a heteromeric GIRK1/2 channel selective activator, ML297 was developed [6]. ML297 is a potent activator of the GIRK1/2 subunit combination having approximately 8-fold selectivity as compared to GIRK1/4 and has no activity towards a number of other potassium channels which show very limited expression in the brain [7]. Selective activation of GIRK channels by ML297 requires two amino acids specific to the GIRK subunit [8]. Systemic application of ML297 has been reported to be associated with anxiolytic [8] and antiepileptic [9] effects in mice. However, whether ML297 has an antinociceptive effect has not been elucidated.

In the present study, we determined first the antinociceptive effect of ML297 in rats using behavioral tests, then used substantia gelatinosa (SG) neurons of spinal cord slices and whole-cell patch-clamp technique to determine the cellular and molecular mechanisms of the antinociceptive effect of ML297.

## Materials and methods

All experimental procedures were approved by the Ethics Committee on Animal Experimentation at Kyushu University (permit no. A28-123-0, A28-124-0), Japan. The work was performed in accordance with the ARRIVE guidelines and the Guidelines of the Japanese Physiological Society.

### Intrathecal catheter implantation and drug administration

The experiments were conducted in 5-week-old male Sprague-Dawley rats. A polyethylene lumbar intrathecal catheter (PE 10, 0.61 mm OD; Intramedic, Clay Adams, Becton Dickinson and Company, NJ) was inserted between the L5 and L6 vertebrae and advanced 3.5 cm rostrally under isoflurane (2–3% in O_2_) anesthesia, as described in detail previously [10]. The proper location of the tip of the catheter was tested 24 h before the pharmacological experiments by assessing the motor blockade following intrathecal injection of 30 μl of lidocaine (50 mg/ml).

### Estimation of the mechanical threshold

To test the effect of ML297 on mechanical nociception, the nociceptive flexion reflex was measured using an analgesy-meter (model 37215; Ugo Basile, Gemonio, Italy) in rats, as described in detail previously [11]. Increasing mechanical pressure was linearly applied through a plastic tip to the hind paw until the rats attempted to withdraw the paw. The threshold measurements were repeated twice, and the average was taken. The effects of ML297 and intrathecally administered vehicle were assessed repeatedly for 35 min post-injection. This yielded an estimation of the mechanical paw withdrawal threshold (PWT).

### Rotarod test

The effect of ML297 on motor performance was assessed using an accelerating rotarod (model 47700; Ugo Basile) as described in detail previously [12]. Briefly, rats were placed on a rotating drum with a speed increasing from 4 to 40 rpm over 5 min. The rats were forced to make forward-walking movements to avoid falling. The latency to fall was measured. The training sessions were performed for two days before the experiments, with three trials on each day. On the experimental day, a baseline response was obtained first, and subsequent measurements were performed for 30 min after intrathecal administration of either ML297 or vehicle.

### Spinal cord slice preparation

Adult rat spinal cord slice preparation was obtained as described in detail previously [13]. Briefly, male Sprague-Dawley rats (5–8 weeks old) were anesthetized with intraperitoneal urethane (1.2–1.5 g/kg), and lumbosacral laminectomy was performed. The spinal cord at the spinal level L1–S3 was removed and placed in pre-oxygenated cold Krebs solution. Immediately after spinal cord removal, the rats were administered with an overdose of urethane and sacrificed by exsanguination. The dura mater, ventral roots, dorsal roots, and the pia-arachnoid membrane of the spinal cord were removed carefully. The spinal cord was mounted on a microslicer (PRO 7; Dosaka Co., Kyoto, Japan), and a transverse slice (600 μm thick) was cut at the spinal level of L3 or L4. The slice was placed into the recording chamber and perfused with Krebs solution (NaCl, in 117 mM; KCl, 3.6; CaCl_2_, 2.5; MgCl_2_, 1.2; NaH_2_PO_4_, 1.2; NaHCO_3_, 2.5; and glucose, 11) saturated with 95% O_2_ and 5% CO_2_ at a rate of 15–20 mL/min and maintained at 36±1°C.

### Patch-clamp recordings from spinal cord slice preparations

Patch electrodes were pulled from thin-walled borosilicate glass capillaries (1.5 mm OD; World Precision Instruments, Sarasota, FL) using a mechanical puller (p-97; Sutter Instrument, Novato, CA). The patch electrodes were filled with potassium gluconate-based internal solution (potassium gluconate, in mM, 136; KCl, 5; CaCl_2_, 0.5; MgCl_2_, 2; EGTA, 5; HEPES, 5; and ATP-Mg, 5; pH 7.2). A patch electrode with a resistance of 8–12 MΩ was advanced at 30–45° angle into the substantia gelatinosa (lamina II), which was visually identified as a distinct translucent band across the superficial dorsal horn under a dissecting microscope with transmitted illumination. After the whole-cell configuration was established, holding potential of -70 mV was applied to record excitatory postsynaptic currents (EPSCs).

To determine the current-voltage relationships of ML297-induced currents, triangle voltage ramp commands (ramp up and ramp down of 0.9 sec duration each) from the -70 mV holding potential (between approximately -50 and -110 mV) were applied every 60 sec during the recording. Current responses to triangle voltage ramps before drug application were subtracted from those during the application. The resulting current ramps were plotted as a function of membrane potential and further analyzed. Miniature excitatory postsynaptic currents (mEPSCs) were analyzed in the presence of 1 μM tetrodotoxin (TTX), a sodium channel blocker. All signals were collected using a patch-clamp amplifier (Axopatch 200B, Axon Instruments, Union City, CA) and digitized with an A/D converter (Digidata 1322A, Axon Instruments). Data were stored on a personal computer using the pCLAMP data acquisition program (version 10.2, Axon Instruments). The frequency and amplitude of mEPSCs were analyzed using a software package (Mini Analysis, version 6.0.3; Synaptosoft Inc., Decatur, GA). The detection criteria for mEPSC events included a threshold amplitude of 4.5 pA, a fast rise time, and a decay curve that approximated exponential decay.

### Reagents

ML297 and [D-Ala(2),N-Me-Phe(4),Gly(5)-ol]-enkephalin (DAMGO) were purchased from Abcam (London, UK). ATP-Mg was purchased from Sigma-Aldrich (St. Louis, MO). Lidocaine was purchased from Aspen (Greeley, CO). Isoflurane was purchased from Pfizer (New York, NY). All other drugs were obtained from Wako (Osaka, Japan).

### Drug application into spinal cord slice preparation

Drugs were dissolved in Krebs solution and applied by perfusion via a three-way stopcock without any change in either the perfusion rate or temperature. The drugs used in this study were ML297, TTX, and naloxone hydrochloride. ML297 was dissolved in dimethyl sulfoxide (DMSO) at a concentration of 10 mM. Although, the high concentration of DMSO in which ML297 was dissolved might be toxic, it has been previously reported that up to 1% DMSO had no adverse effect on the cell response in pituitary and cardiac cultured cell lines [14] or spinal cord slice preparations [15]. Therefore, in the present study, ML297 was used at concentrations of up to 100 μM from a 10 mM stock. The other drugs were dissolved in distilled water. All drugs were diluted to their final concentration in Krebs solution immediately before use.

### Statistical analyses

All continuous variables are expressed as mean±standard error of the mean (SEM). Data were analyzed with Prism 6 (GraphPad Software, La Jolla, CA). A P-value of <0.05 was considered statistically significant. In the behavioral experiments, comparisons of drug effects over time were performed using two-way repeated measures ANOVA, with time and drug treatment as within- and between- subjects’ factors, respectively. When the results of two-way ANOVA were significant, *post hoc* comparison between the drug treatment groups and the vehicle at individual time points were tested using the Bonferroni adjustment for multiple comparisons. The sample size in the behavioral study was calculated based on a previous study [11]. We assumed >50% prolongation of paw withdrawal latency in response to tactile stimuli as an effective response to the analgesic as compared to the vehicle. This value represents the mean difference and the standard deviation was about 15%. The sample size calculation indicated that six rats were required in each group with an alpha of 0.05 and a power of 0.9 based on two-sided tests. For the electrophysiological results, n refers to the number of neurons studied. The Student’s paired t test was used to compare the amplitude of outward currents. To determine the concentration-response relationship of ML297, multiple comparisons were performed using the Student’s paired t test with Bonferroni correction, and a P-value of <0.0167 was considered statistically significant. Cumulative probability plots were constructed for mEPSC amplitude and frequency, which were compared using Kolmogorov–Smirnov test.

## Results

### Antinociceptive effect of ML297 on mechanical nociception

**Fig 1:**
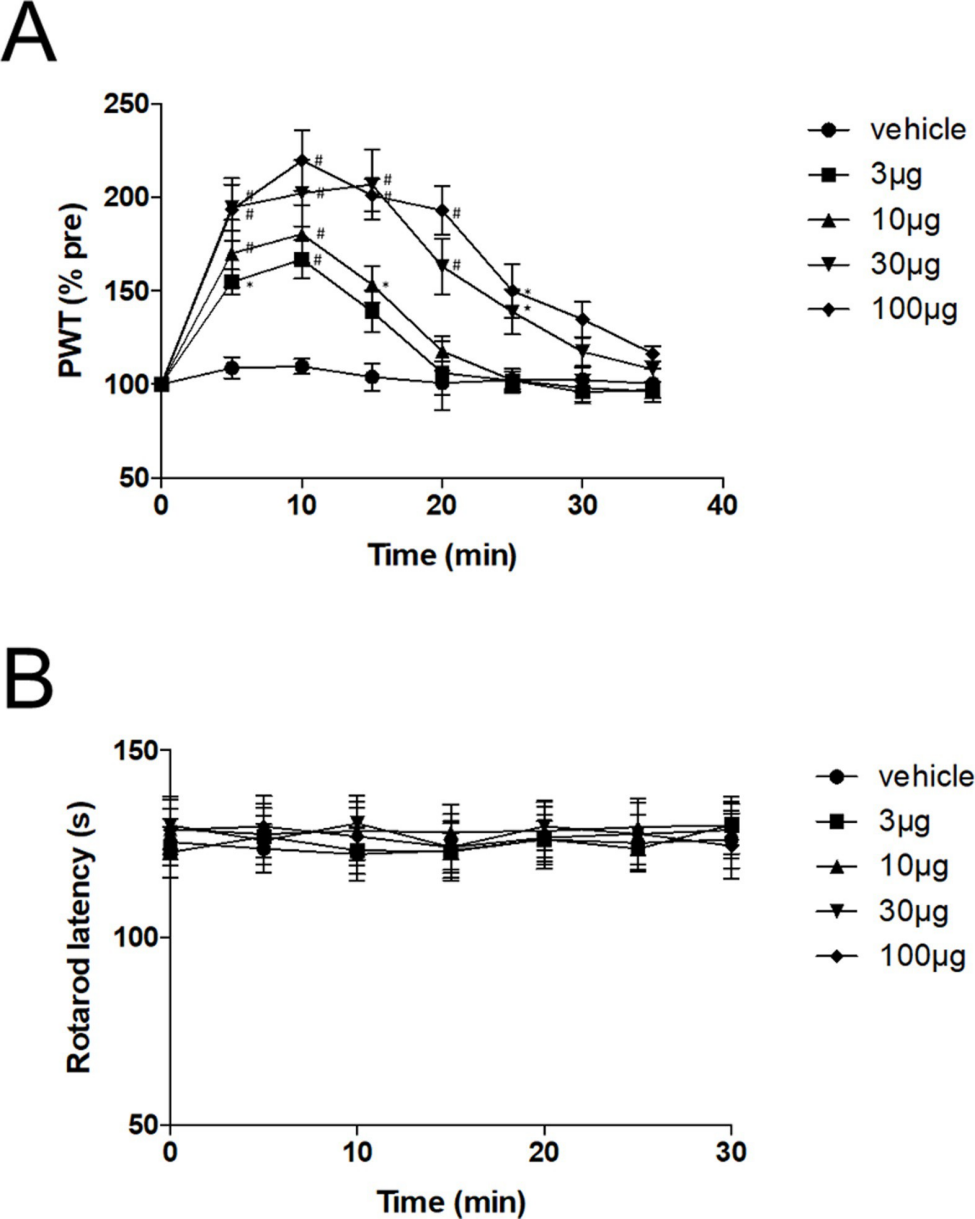
Effects of intrathecal administration of ML297 on mechanical nociception. (A) ML297 increased the paw withdrawal threshold in a dose-dependent manner. Significant effects were seen at 3 μg and higher doses. Data are mean±SEM of the pre-injection baseline levels (n = 6 rats/group). *P<0.01, ^#^P<0.001, compared to the vehicle at corresponding time points. (B) Effects of intrathecally administered ML297 on motor performance in the rotarod test. ML297 did not affect motor performance at concentrations up to 100 μg. Data are mean±SEM (n = 6 rats/group).

We first assessed whether ML297 modulates mechanical nociception by using the paw pressure test. The paw withdrawal latency was assessed every 5 min after intrathecal administration of ML297. As shown in Fig 1A, ML297 prolonged the paw withdrawal latency in a dose-dependent manner. The mechanical withdrawal threshold reached a peak at ~10 min and decreased to the vehicle level at ~35 min after ML297 administration.

### Effects of ML297 on motor function

Since impaired motor function could explain the prolonged paw withdrawal latency, we next examined the effect of intrathecal administration of ML297 on motor activity using the rotarod test. The baseline latency was 125±6 sec. ML297 at doses up to 100 μg did not affect the rotarod latency, indicating that the ML297-induced prolongation of the paw withdrawal latency is not related to impaired motor function (Fig 1B).

### Postsynaptic actions of ML297 in spinal cord slice preparation

**Fig 2:**
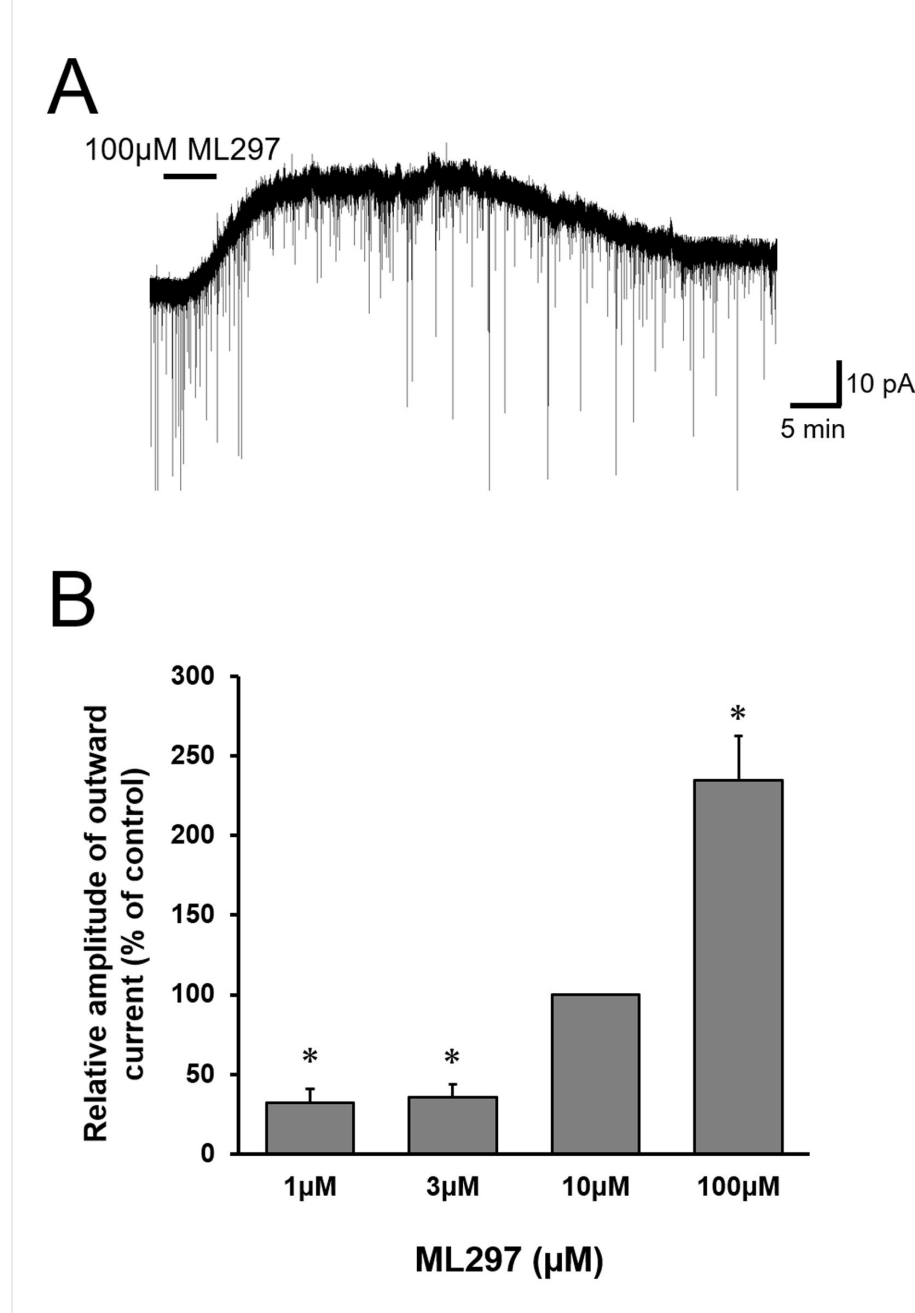
ML297- induced outward current in SG neurons. (A) Representative recording of 100 μM ML297-induced outward current in voltage-clamp mode (V_H_ = -70 mV) in a spinal cord slice preparation. (B) Correlation between the normalized amplitudes of the outward currents induced by ML297 relative to those at concentrations of 10 μM. Data are mean±SEM (n = 5–12). ML297 dose-dependently induced the outward current. *P<0.0167.

To further investigate the antinociceptive effect of ML297, whole-cell patch-clamp recordings in SG neurons using adult rat spinal cord slice preparations were performed. ML297 perfusion for 5 min induced an outward current in all the SG neurons tested (n = 24) in the voltage-clamp mode at a holding potential of -70 mV (Fig 2A). The average amplitude of the peak currents induced by ML297 at concentration of 100 μM was 28.9±5.8 pA (*n* = 8). When examined in the concentration range of 1–100 μM, the amplitude of the ML297-induced outward currents was enhanced as the concentrations increased (Fig 2B).

**Fig 3:**
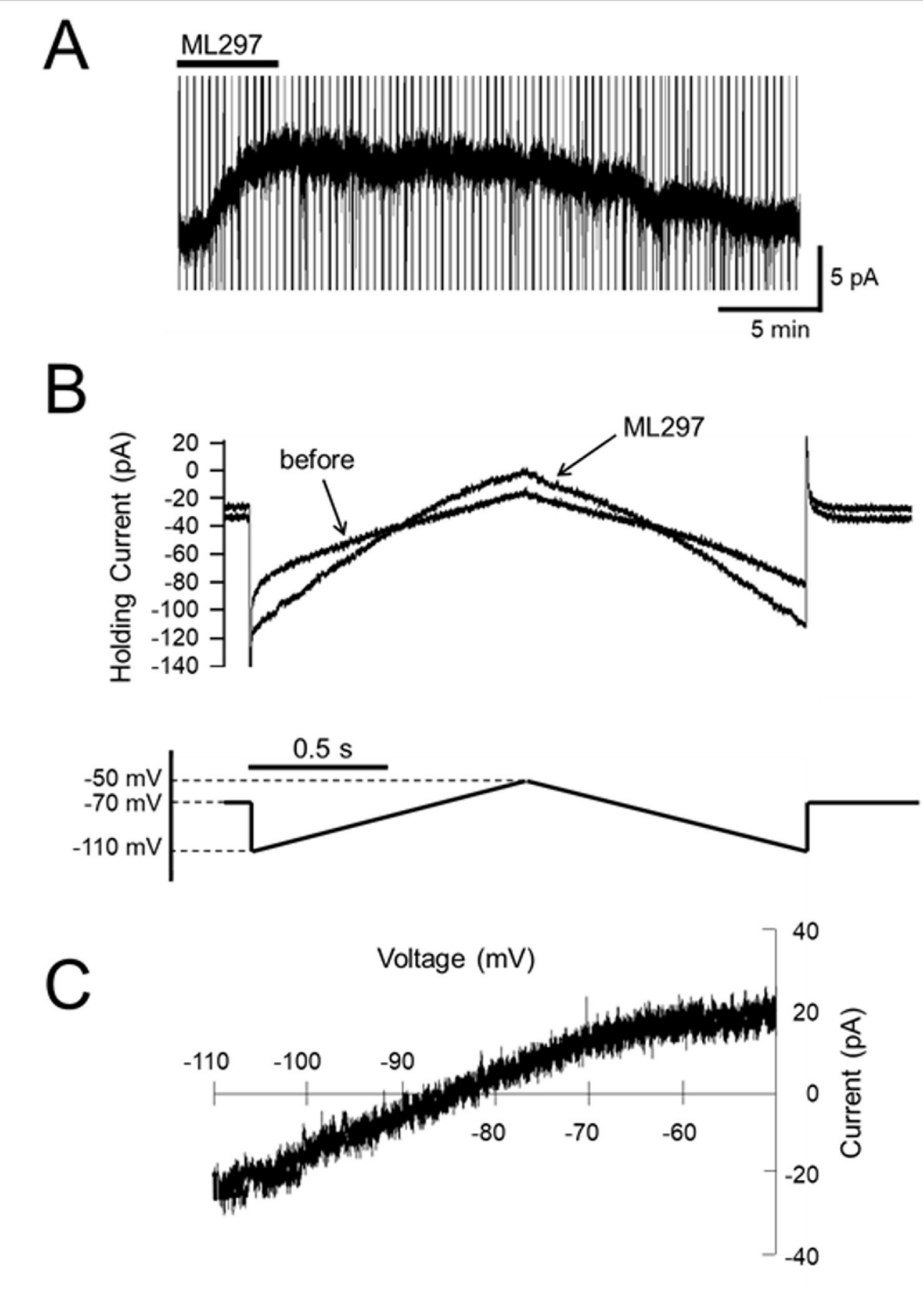
Voltage dependency of ML297-induced current. (A) Representative recording of ML297-induced outward current in voltage-clamp mode (V_H_ = -70 mV) with voltage ramp commands in a spinal cord slice preparation. Vertical lines are traces of the current response to voltage ramp commands. (B) Current response to the voltage ramp (lower trace) over an expanded time base. The current-voltage relationship was recorded at the peak response to ML297 application. (C) Current-voltage relationship. The current-voltage relationship identified that the reversal potential of ML297 was close to that of the other families of potassium channels.

To examine the current-voltage relationship of the ML297-induced current, triangles voltage steps -50 mV to -110 mV and back to -50 mV were applied every 20 sec before and during ML297 application (Fig 3A and 3B). The voltage sensitivity of the ML297-induced current generated by the ascending ramp command was similar to that of the descending command (Fig 3B). After subtraction of the control current, the resulting ramp currents were plotted as a function of the command voltage (Fig 3C). The current-voltage relationships of these ML297-induced currents had a mean reversal potential of -87.9±1.7 mV (n = 5), which was close to the equilibrium potential of other families of potassium channels (-92 mV) calculated from the Nernst equation [16], indicating GIRK channel activities.

### Presynaptic action of ML297 in the spinal cord slice preparations

**Fig 4:**
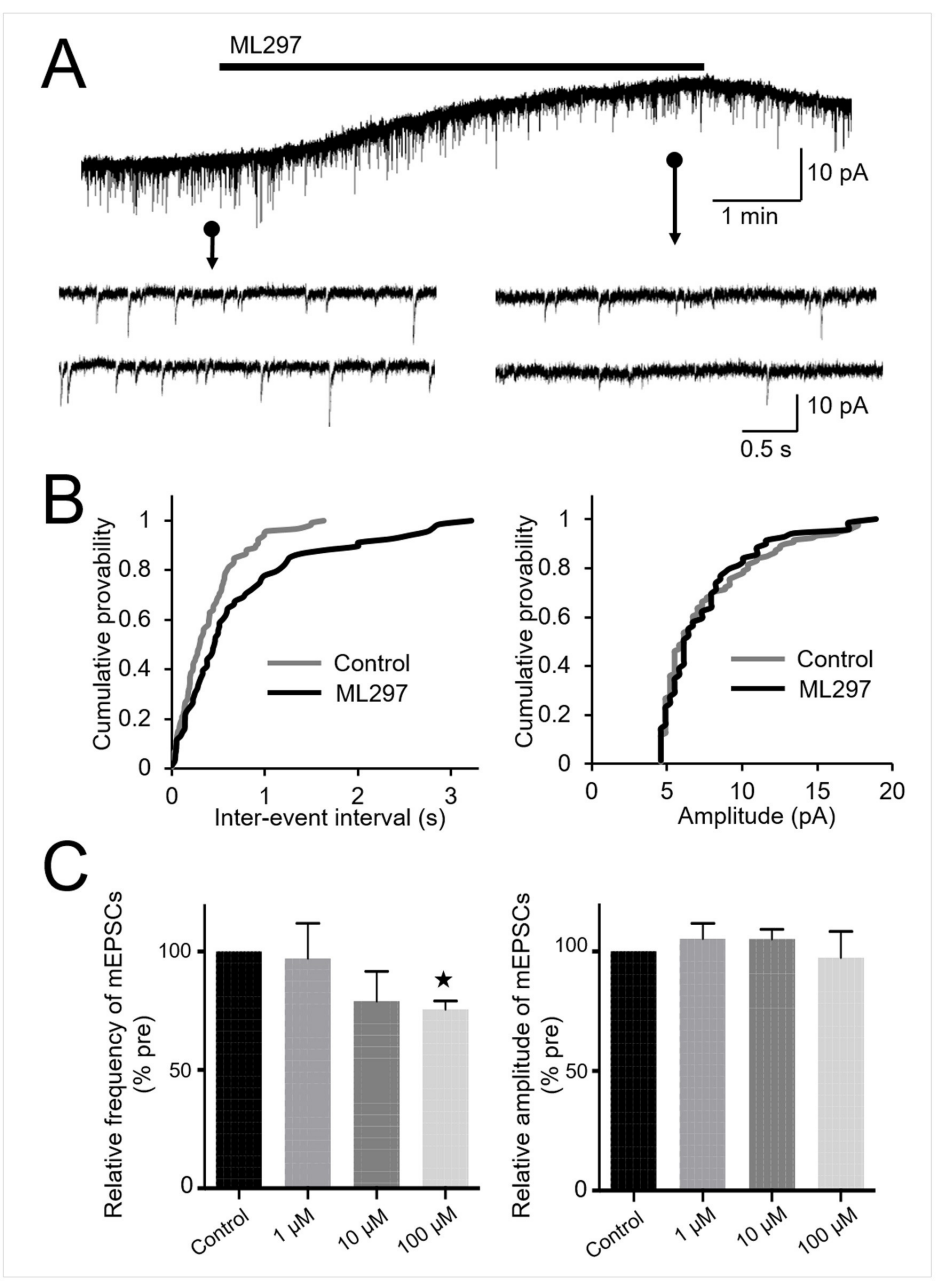
Effects of ML297 on mEPSCs. (A) Representative recording of mEPSCs before (left) and after (right) administration of 100 μM ML297. (B) Cumulative distribution of the inter-event interval and amplitude of mEPSCs recorded before application (grey line) and in the presence (black line) of 100 μM ML297. ML297 did not affect the distribution of the amplitude (P = 0.99, right), but shifted the distribution to a longer inter-event interval (P<0.05, left). (C) Summary of normalized frequencies (left) and amplitudes (right) of mEPSCs relative to the pre-application base line levels. ML297 at 100 μM, but not at lower concentrations of 10 and 1 μM, significantly decreased the frequency of mEPSCs. ML297 had no significant effect on mEPSC amplitude irrespective of the concentration. Data are mean±SEM (n = 8).

We also examined whether ML297 modulates glutamate release from the presynaptic terminals of SG neurons. In the presence of TTX, the frequency of mEPSCs decreased significantly following the application of ML297 at 100 μM (73.7±4.6%, n = 8, P<0.001), but not at 10 μM or 1 μM (Fig 4A–4C). The amplitude of mEPSCs was not affected by 100 μM of ML297 (Fig 4B and 4C). These results suggest that ML297 acts presynaptically, decreasing glutamate release, possibly due to the activity of GIRK channels.

### GIRK channel activation under inhibition of μ opioid receptors

**Fig 5:**
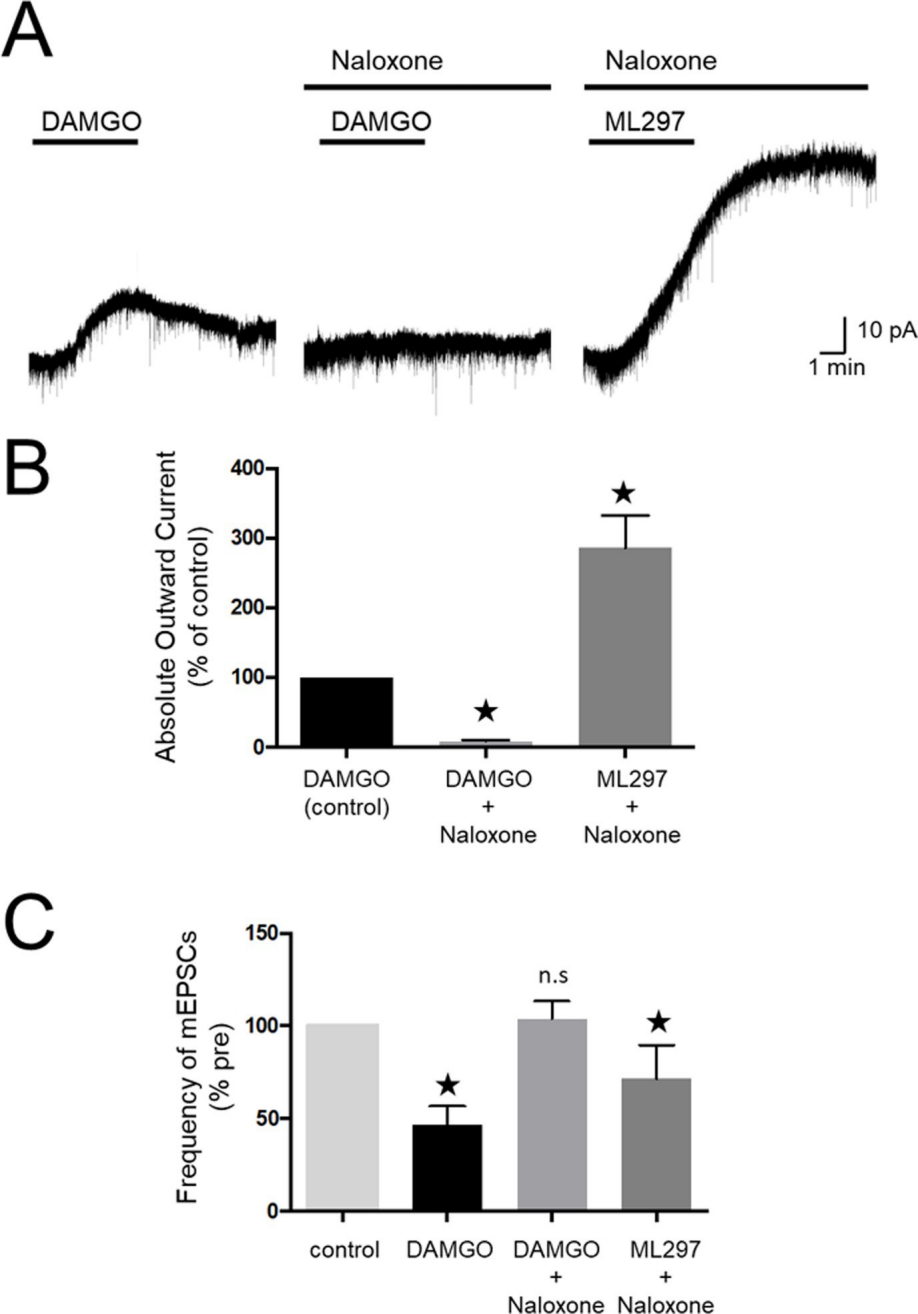
Effects of naloxone on presynaptic and postsynaptic actions of ML297. (A) Representative recording of currents applied by DAMGO in the absence (left) and presence (middle) of naloxone and currents applied by ML297 in the presence of naloxone (right). The readings were obtained from the same neuron (n = 7, V_H_ = -70 mV). (B) Summary of normalized amplitudes of DAMGO and ML297-induced outward currents. The peak of the DAMGO-induced current was set as a reference value (control). (C) Summary of normalized frequencies of mEPSCs for each condition. Data in (B) and (C) are mean±SEM (n = 7, each). ^★^P<0.05, n.s.; not significant.

μ opioid-induced analgesia is known to be mediated by the activation of GIRK channels [17]. However, whether the μ opioid receptor is responsible for major GIRK channel activity in the spinal cord remains to be elucidated. In the patch-clamp recordings of SG neurons, perfusion of 100 μM DAMGO, a selective μ opioid receptor agonist, induced outward currents, which were almost completely blocked in the presence of 100 μM naloxone, a μ opioid receptor antagonist (Fig 5A and 5B). However, ML297-induced outward currents were mostly not suppressed even in the presence of naloxone (Fig 5A and 5B). Furthermore, naloxone reversed the reduction of mEPSCs frequency induced by DAMGO but not ML297 (Fig 5C). These results suggest that ML297 activates both pre- and post-synaptic GIRK channels in the dorsal horn neurons in a μ opioid receptor-independent manner.

## Discussion

By using behavioral studies and whole-cell patch-clamp recordings from spinal lamina II (SG) neurons, we provide evidence in the present study that ML297, a selective GIRK1/2 activator, affects nociceptive transmission in the spinal cord.

Systemic application of ML297 in mice has been reported to have anxiolytic and antiepileptic effect. Specifically, intraperitoneal injection of 30 mg/kg ML297 triggered anxiolysis [8], and 60 mg/kg dose eradicated seizures [9] in mice. Unfortunately, these reports did not discuss the influence of nociception. Since the spinal dorsal horn is rich in GIRK1 and 2 subunits, especially in the outer layers of SG where nociceptive information from the primary afferents are integrated [18], GIRK1/2 activation can be considered a new strategy for pain management. Recently, Abney et al [19]. reported the analgesic efficacy of VU0466551, another GIRK1/2 channel activator, which is 2-fold more potent than ML297. Intraperitoneal injection of 10–30 mg/kg VU0466551 together with subcutaneous injection of submaximally effective doses of morphine, prolonged the latency to heat stimulation, though VU0466551 itself had no analgesic effect on heat stimulation [19]. In the same study, VU0466551 alone induced analgesia against formalin-induced pain. Unfortunately, no explanation was provided for the discrepancy between the effect of VU0466551 on the hot plate assay and the formalin assay. In the present study, unlike the above report, we demonstrated that intrathecal, but not systemic, administration of ML297 had antinociceptive effect without impairing motor function in rats. The discrepancy between our results and those of the above study could be due to differences in the potency, efficacy, selectivity, drug metabolism, and/or pharmacokinetic properties of the drugs used (ML297 vs. VU0466551). It could be also due to the difference in the route of administration (intrathecal vs. intraperitoneal), species difference (rat vs. mouse), and/or difference in type of nociception studied (mechanical vs. thermal). Nevertheless, on the grounds that the nociceptive transmission can be modulated by a variety of endogenous systems in the spinal dorsal horn and that intrathecal drug delivery is clinically used for the treatment of various pain sensations [20], intrathecal ML297 is a potentially promising strategy for acute pain therapy.

To further assess the underlying antinociceptive mechanism of ML297, we conducted patch-clamp experiments. ML297 induced membrane hyperpolarization of SG neurons probably through efflux of potassium ions in the postsynaptic dorsal horn neurons. We also showed that ML297 acts presynaptically in SG neurons, which was demonstrated by the decrease in mEPSC without affecting its amplitude, indicating decrease in presynaptic glutamate release. Another patch-clamp study showed similar outward current evoked by ML297 in lamina I projection neurons and GABAergic interneurons in the superficial dorsal horn neurons of adult female mice [21]. Since both lamina I and II represent major targets of the nociceptive primary afferents and play a role in modulating and transmitting incoming sensory information, these results add further support for the feasibility of ML297 in pain relief.

Although we did not define the value of the effective concentration producing half-maximal response (EC_50_) of ML297 on our rat spinal slice preparations, the concentrations of ML297 we used were much higher than those used in previous reports where it has been suggested that the EC_50_ of ML297 was 0.2 μM in GIRK1/2 expressing HEK293 cells and 0.3 μM in cultured hippocampal cells [8]. The higher concentrations of ML297 required in our study might be explained by the low tissue permeability of ML297 into the SG neurons recorded in the slice preparations.

As the name suggests, GIRK channels are effectors of G_i/o_ GPCRs, including μ-opioid, GABA_B_, dopamine D_2_, serotonin 5-HT_1A_, adenosine A_1_, acetylcholine M_2_, and adrenergic α_2_ receptors. The G_βγ_ subunits of these G proteins activate GIRK channels resulting in reduced cellular excitability [22]. Immunohistochemical analysis of mouse SG neurons conducted by another group showed that GIRK1/2-containing channels are colocalized with the opioid receptors [23]. Another group reported that GIRK channels were indispensable for peripheral opioid analgesia [24]. Notably, they demonstrated that GIRK1 and GIRK2 expression was absent from mouse peripheral sensory neurons, but present in those of human and rat [24]. It has also been reported that intrathecally administered morphine evoked analgesia and thermal antinociception through the GIRK1/2-containing channels [18]. These results suggest that the antinociceptive effect of ML297 observed in the present study could be dependent on the opioid receptor. However, in the present study, we demonstrated that ML297 opens postsynaptic GIRK1/2 channels in the SG neurons and produces hyperpolarization by the efflux of potassium ion even in the presence of naloxone, a μ opioid antagonist, indicating a μ opioid receptor–independent mechanism. The results of another electrophysiological study using HEK293 cells expressing GABA_B_ receptor and GIRK1/2 channels also suggested that ML297 directly activates GIRK1/2 channels without requiring GPCRs [6], and some other drugs (e.g., ethanol, volatile anesthetics, and naringin) are also reported to activate GIRK channels in a G-protein–independent manner [8]. Since many GPCRs play important roles in biological functions, the direct action of ML297 on GIRK channels makes it a promising analgesic in the sense that pain relief can be achieved with little adverse effects.

Opioid tolerance represents reduction in effect following prolonged use with loss of drug potency. The proposed molecular and cellular mechanisms of opioid tolerance include those mediated by the internalization [25, 26] and desensitization [27, 28] of opioid receptors after long exposure to opioid. In the present study, we found that ML297 produced outward current by directly activating GIRK channels even in the presence of naloxone, which blocked μ opioid-induced outward current. These results suggest that ML297 could exert antinociceptive effect under inhibition of μ opioid receptors, although the administration of naloxone did not fully mimic the opioid receptor internalization or desensitization leading to opioid tolerance. Therefore, ML297 is also a potentially useful antinociceptive drug for patients with drug tolerance under chronic administration of opioid.

In this regard, common cellular mechanisms have been suggested for the transition from acute to chronic pain, and development of μ-opioid receptor tolerance [29]. Accordingly, we believe that ML297 can also be potentially useful for chronic pain.

In conclusion, the present study demonstrated an antinociceptive action for intrathecally administered ML297 in rats through the activation of pre- and post-synaptic GIRK channels in the spinal cord. These findings provide promising opportunities for the development of new therapies for chronic pain and opioid tolerance.
